# Combination of graphene oxide and platelet-rich plasma improves tendon–bone healing in a rabbit model of supraspinatus tendon reconstruction

**DOI:** 10.1093/rb/rbab045

**Published:** 2021-08-04

**Authors:** Dingsu Bao, Jiacheng Sun, Min Gong, Jie Shi, Bo Qin, Kai Deng, Gang Liu, Shengqiang Zeng, Zhou Xiang, Shijie Fu

**Affiliations:** 1 Department of Orthopedics, Affiliated Traditional Chinese Medicine Hospital of Southwest Medical University, Luzhou, Sichuan 646000, PR China; 2 Department of Orthopedics, West China Hospital, Sichuan University, Chengdu, Sichuan 610041, PR China; 3 Department of Orthopedics, Hospital of Chengdu University of Traditional Chinese Medicine, Chengdu, Sichuan 610075, PR China

**Keywords:** platelet-rich plasma, graphene oxide, controlled release, rotator cuff tear, tendon–bone interface healing

## Abstract

The treatment of rotator cuff tear is one of the major challenges for orthopedic surgeons. The key to treatment is the reconstruction of the tendon–bone interface (TBI). Autologous platelet-rich plasma (PRP) is used as a therapeutic agent to accelerate the healing of tendons, as it contains a variety of growth factors and is easy to prepare. Graphene oxide (GO) is known to improve the physical properties of biomaterials and promote tissue repair. In this study, PRP gels containing various concentrations of GO were prepared to promote TBI healing and supraspinatus tendon reconstruction in a rabbit model. The incorporation of GO improved the ultrastructure and mechanical properties of the PRP gels. The gels containing 0.5 mg/ml GO (0.5 GO/PRP) continuously released transforming growth factor-β1 (TGF-β1) and platelet-derived growth factor (PDGF)-AB, and the released TGF-β1 and PDGF-AB were still at high concentrations, ∼1063.451 pg/ml and ∼814.217 pg/ml, respectively, on the 14th day. *In vitro* assays showed that the 0.5 GO/PRP gels had good biocompatibility and promoted bone marrow mesenchymal stem cells proliferation and osteogenic and chondrogenic differentiation. After 12 weeks of implantation, the magnetic resonance imaging, micro-computed tomography and histological results indicated that the newly regenerated tendons in the 0.5 GO/PRP group had a similar structure to natural tendons. Moreover, the biomechanical results showed that the newly formed tendons in the 0.5 GO/PRP group had better biomechanical properties compared to those in the other groups, and had more stable TBI tissue. Therefore, the combination of PRP and GO has the potential to be a powerful advancement in the treatment of rotator cuff injuries.

## Introduction

In recent years, the incidence of rotator cuff tears caused by sports, aging and car accidents has increased dramatically. The injuries of rotator cuff tear can result in shoulder pain and limited motor function. Statistically, 5–40% of the general population have been diagnosed with a rotator cuff tear, and the prevalence of such injuries is steadily increasing [1]. The clinical treatments for rotator cuff tears include conservative treatments such as corticosteroids injection, mechanical conditioning and orthotics, and surgical interventions using autologous and allogeneic grafts. However, the treatment results are often unsatisfactory. The difficulty in treating rotator cuff tear is related to the healing of the tendon–bone interface (TBI) [2, 3]. Due to its complex four-layer structure and lack of blood supply, the TBI is usually repaired with scar tissue, which is associated with poor force transmission [4, 5]. Consequently, the re-tear rate is extremely high (8–94%) [6]. Currently, tissue engineering is considered to be one of the most effective methods of treating rotator cuff tears [7].

As we all know, there are three key elements in tissue engineering, including scaffolds, cells and growth factors (GFs). For the construction of tissue engineering tendons, the scaffold design for the complex TBI structure and the cost of multiple involved GFs are the main challenges. Moreover, the transplantation of cells, such as stem cells, may lead to tumorigenesis. Recently, platelet-rich plasma (PRP) that has the potential to solve these problems has aroused the interest of researchers. PRP is a type of platelet concentrate obtained by the centrifugation of an autologous whole blood sample, with a simple preparation process and a low immune response [8, 9]. It has a rich source of GFs, such as transforming growth factor-β1 (TGF-β1), platelet-derived growth factor (PDGF), vascular endothelial growth factor, which perform functions including regulating inflammation, promoting cell proliferation and differentiation, and accelerating angiogenesis [10–12]. For these reasons, PRP has been repeatedly investigated for its potential to accelerate rotator cuff repair; however, there is some debate surrounding its efficacy. A study from Wang *et al*. showed that PRP injection effectively improved the short-term outcomes of full-thickness rotator cuff tears, while Beck *et al*. demonstrated that augmenting rotator cuff repairs with PRP failed to enhance TBI healing and biomechanical properties of the repaired tendons [6, 13]. Possible reasons for the conflicting findings include differences in the preparation methods and the form of the PRP product (solution or gel) which may affect the GF components, and the resulting short half-life of the GFs which may weaken the therapeutic effect [9, 14]. Thus, improving PRP products would likely enhance its therapeutic effect on rotator cuff tears.

Graphene oxide (GO) is one of the thinnest materials in the world and is widely used in tissue engineering [15, 16]. GO modification may improve the mechanical properties of biomaterials and promote cell proliferation and differentiation [17, 18]. Additionally, as GO has a large specific surface area, numerous functional groups and excellent hydrophilicity, it is often used as a drug carrier or GF delivery system based on covalent, non-covalent and electrostatic bonding [19–21]. Therefore, we expect that GO, when incorporated into a PRP product, will act as a sustained GF release system, improve the mechanical properties of the PRP gel and ultimately promote rotator cuff regeneration.

In this study, we developed a GO/PRP gel for the treatment of rotator cuff tears. The effects of the gel on the proliferation and differentiation of bone marrow mesenchymal stem cells (BMSCs) were evaluated *in vitro*. Surgical tears were created in the supraspinatus tendon of rabbits and then treated with surgical suturing and the GO/PRP gel. We hope that our work will provide a reference for future research on rotator cuff repair and clinical treatment of tendon injuries.

## Materials and methods

The whole experimental protocol was approved by the Animal Care and Experiment Committee of the Affiliated Traditional Chinese Medicine Hospital of Southwest Medical University (2020680). All the reagents were purchased from SigmaAldrich (USA) and all cell culture media were supplied by HyClone (USA) or Cyagen (Guangzhou, China) unless otherwise stated.

### Preparation and characterization of GO/PRP gels

#### PRP preparation

PRP was obtained from the whole blood of rabbits (New Zealand white rabbit; male; 24 weeks old; *n* = 12) using the double-centrifugation method [22]. Briefly, 10 ml of blood was collected from the rabbit’s ear artery and anti-coagulated with 1 ml 10% (w/v) sodium citrate. After the first centrifugation, at 200 *×g* for 10 min at 25°C, the upper plasma layer and the middle buffy coat were transferred to a new sterile tube. After the second centrifugation, at 250 *×g* for 10 min at 25°C, the supernatant was discarded and the lower portion (∼2.0 ml) of PRP was retained and transferred to another sterile tube. Platelet counting was then performed on the original whole blood sample and the obtained PRP.

#### Preparation of GO/PRP gels

A single layer of GO powder with an average diameter of 10 μm (TimesNano, Chengdu, China) was sterilized using ethylene oxide and used to prepare a solution with phosphate buffer solution (PBS) via ultrasonic dispersion for 2 h. The GO solution and PRP were mixed at a volume ratio of 1:2, and the resulting suspension was stirred for 2 h at room temperature. Next, 1 ml of the obtained GO/PRP mixture was added into a 48-well plate and 80 µl bovine thrombin solution (10% (w/w) calcium chloride solution containing 1 U/µl bovine thrombin) was pipetted into each well to form a gel. The gelification took 30–60 s. The final gels contained different concentrations of GO—either 0.25, 0.5 or 1 mg/ml—and were labeled 0.25 GO/PRP, 0.5 GO/PRP and 1.0 GO/PRP, respectively. The pure PRP gels containing no GO were labeled PRP.

#### Degradation detection

For the *in vitro* degradation test, the surfaces of the gels were dried with absorbent paper and weighed (M0). The gels were transferred into 15 ml tubes and incubated in simulated body fluid at 37°C on a shaking table at 100 r min^−1^. At defined time points (1, 7 and 14 days), the gels were collected and their surface were dried with absorbent paper before they were reweighed (M1). The degradation was measured as (M0 – M1)/M0 × 100%.

#### Ultrastructure and porosity analysis

The gels were refrigerated at −20°C for 2 h, quickly transferred to refrigeration at −80°C overnight, then lyophilized. The samples were cut and prepared with a surgical blade. After gold spraying, the surface morphology of the samples was observed using a ZEISS EVO 10 scanning electron microscope (SEM). The pore size and porosity of the freeze-dried gels were calculated as previously reported [23].

#### Detection of GF release

The gels were placed into 15 ml tubes and incubated in 2 ml PBS at 37°C on a shaking table at 100 r min ^–1^. At different time points (1 h, 12 h, 24 h, 3 days, 5 days, 7 days and 14 days), the PBS solution was collected and stored at –20°C, and 2 ml of new PBS solution was added in replacement. At each time point, the concentrations of TGF-β1 and PDGF-AB in the collected PBS were quantified using corresponding enzyme-linked immunosorbent assay kits (Elabscience, China).

#### Elastic modulus estimation

The elastic modulus of the gels was evaluated using compressive testing. A dynamic thermomechanical analysis (Q-800, TA, USA) instrument was used to test the stress/strain of the samples. Gels were compressed using 0.01 N of force at a loading velocity of 1 mm/min until a final displacement of 2 mm was reached.

### Evaluation of gel biotoxicity and capacity to promote BMSC proliferation and differentiation

#### Cell proliferation

BMSCs at passage 2 (ZQ0682) were purchased from Zhongqiaoxinzhou Biotech (Shanghai, China). Cells were cultured with low-glucose Dulbecco’s modified Eagle’s medium (DMEM) containing 10% fetal bovine serum (Gibco, USA) and 1% (v/v) penicillin–streptomycin solution (Solarbio, China). Cells at passage 3 were used in this study: 2 × 10^4^ BMSCs were seeded in the lower chamber of a 24-well Transwell plate (pore size: 0.4 μm; Corning, USA). After 4 h of inoculation, most of the cells adhered to the plate and gels were placed in the upper chamber. Cells cultured without gels were labeled Blank. The medium was changed every 2 days. A Cell Counting Kit-8 (CCK-8; Dojindo, Japan) test was performed according to instructions after culturing for 1, 3 and 5 days.

#### Cell viability

The gels were placed in a 48-well plate and soaked in low-glucose DMEM for 24 h. The medium was removed, and 1 ml of BMSC medium containing 1 × 10^5^ cells was seeded onto each gel before incubation. The culture medium was replaced every 2 days. After 5 days of culture, the gels were stained using the live–dead staining kit (Solarbio), according to the manufacturer’s instructions. The stained samples were observed under a laser scanning confocal microscope (Nikon, Japan).

#### Osteogenic- and chondrogenic-related gene expression

To evaluate the *in vitro* osteogenic and chondrogenic differentiation of BMSCs on the gels, the 0.5 GO/PRP gel, which had low biotoxicity and significantly promoted cell proliferation, was selected for follow-up experiments, with the PRP gel as the control. The gels were placed in a 48-well plate and 1 × 10^5^ BMSCs were seeded onto each gel. The cells were cultured in BMSC medium for 3 days, followed by a period in either osteogenic or chondrogenic induction medium (Cyagen, China), as follows. After 3, 7 and 14 days of osteogenic induction, quantitative reverse transcription polymerase chain reaction (qRT-PCR) was performed to analyze the osteogenic-related gene expression of the samples, as previously described [24]. After 14 and 21 days of chondrogenic induction, chondrogenic-related gene expression was analyzed using the same method. Osteogenic-related genes included *Runx2 and OCN*, and chondrogenic-related genes included *Sox9 and Col II*. Glyceraldehyde 3-phosphate dehydrogenase was used as an internal control. All primers were synthesized by Sangon Biotech (Shanghai, China; Table 1).

**Table 1.** Primer sequences used for RT-PCR

Gene	Primer/probe	Sequence
*OCN*	Forward	GCTCAGCCTTCGTGTCCA
	Reverse	CCTGCCCGTCGATCAGTT
*Runx2*	Forward	CCGTCCATTCACTCCACCAC
	Reverse	GAAGACTGGGAGTCCAAGGTG
*Sox9*	Forward	CCGGACTACAAGTACCAGCC
	Reverse	GAATGGACCTCGCTCATGCC
*Col II*	Forward	CTGCAGCACGGTATAGGTGA
	Reverse	AACACTGCCAACGTCCAGAT
*GAPDH*	Forward	TGGAATCCACTGGCGTCTTC
	Reverse	TCATGAGCCCCTCCACAATG

#### Immunofluorescence staining of OCN and Col II

After 14 days of osteogenic induction and 21 days of chondrogenic induction, immunofluorescence staining of OCN and Col II were performed, respectively. Samples were fixed with 4% paraformaldehyde for 30 min, treated with 0.5% Triton X-100 for 15 min and blocked with 2% BSA (Solarbio) for 40 min. After washing with PBS, the samples were stained against an OCN primary antibody (1:100; GeneTex, USA) or a Col II primary antibody (1:100; Proteintech, China) overnight. The cells were then incubated in the secondary antibody of goat anti-mouse IgG H&L, Alexa Fluor 594 or 488 (1:200; Zen BioScience, China) for 2 h at 37°C, and the nuclei were stained with DAPI (Solarbio). The stained samples were observed under the laser scanning confocal microscope.

### Repairing rotator cuff tears in rabbits

#### Animal surgery

Seventy-two 6-month-old male New Zealand white rabbits (2.5–3 kg) were randomly divided into three groups. For rabbits in all groups, a lateral rotator cuff tear model was created. Briefly, the rabbits were anesthetized using sodium pentobarbital. A lateral skin incision between the acromion and greater tuberosity was made, followed by deltoid muscle retraction to expose the supraspinatus tendon. The supraspinatus tendon was cut at the base of the tendon insertion using a sharp scalpel blade. Two bone tunnels were created at the articular margin of the footprint to the lateral humeral cortex. For rabbits with gel implantation, an extra 0.5 cm of tendon was removed from the detached tendon at the isolated end. PRP gel or 0.5 GO/PRP gel was placed at the TBI area (on the footprint and below the detached tendon). The tendon was then reattached to the gel or footprint, with 2-0 Ethibond sutures passed through the bone tunnels and tied. The wound was closed layer by layer. Each rabbit was allowed to perform normal cage activities without restriction. Rabbits implanted with PRP gel or 0.5 GO/PRP gel were labeled PRP or GO/PRP, respectively. Rabbits treated without implantation (surgical suturing only) were labeled Blank.

#### Magnetic resonance imaging and micro-computed tomography analysis

To evaluate supraspinatus tendon repair, magnetic resonance imaging (MRI) (Achieva 3.0T; Philips, The Netherlands) scanning was performed after the rabbits in each group underwent anesthesia at 8 and 12 weeks postoperatively, as previously described [25]. Signal-to-noise quotient was used to quantify the signal intensity of the healing tissue, and was defined as [signal (supraspinatus tendon-bone junction) – signal (long head of the biceps tendon)]/signal (background). After 8 and 12 weeks post surgery, the rabbits were sacrificed with excessive anesthetic, and the supraspinatus tendon and the humeral head of each rabbit was harvested and fixed in 10% formalin for micro-computed tomography (μCT) (SkyScan 1176; SkyScan, Aartselaar, Belgium) imaging. Images were reconstructed using the system software and the region of interest (4.5 mm in the *x*-axis, 4.5 mm in the *y*-axis, 3 mm in the *z*-axis) was positioned on the supraspinatus TBI area [26]. Bone volume fraction [bone volume (BV)/tissue volume (TV)], mean trabecular thickness (TbTh) and mean trabecular spacing (TbSp) were calculated.

#### Histological evaluations

The hearts, livers, lungs and kidneys of rabbits sacrificed at 12 weeks of implantation were harvested and stained with hematoxylin and eosin (H&E) to evaluate the toxicity response of gels implanted *in vivo*. The greater tubercle of the humeral head with attached supraspinatus tendon of each rabbit was harvested at 8 and 12 weeks post surgery. Specimens were fixed with 10% neutral formalin for 24 h and decalciﬁed for 4 weeks. The specimens were trimmed, dehydrated and embedded in paraffin. Longitudinal sections 5 μm thick were cut in the coronal plane and stained with H&E, Masson’s trichrome (Masson) and picrosirius red.

#### Biomechanical tests

At 8 and 12 weeks post implantation, biomechanical evaluations of the obtained supraspinatus tendon and the humeral head of each rabbit were performed with a material testing machine (Electron Puls E3000; Instron, USA). The sample was fixed into the machine in the anatomical posture, to allow the tensile loading of the tendon in the anatomical direction [27]. The biomechanical properties of the tendons were evaluated by the load to failure at a rate of 10 mm/min, with a preload of 5 N after 10 consecutive preconditioning loads.

### Statistical analysis

All experiments were performed in triplicate, unless otherwise indicated. Data were expressed as means ± standard deviations. Statistical analysis was performed using SPSS Statistics 16.0 (Chicago, IL, USA) using one-way analysis of variance, followed by the Tukey’s multiple-comparison test to evaluate between-group differences. *P* < 0.05 was considered statistically significant.

## Results

**Figure 1.**
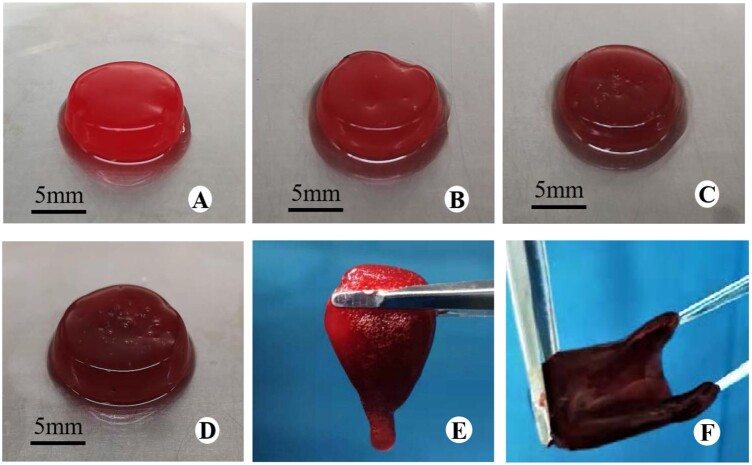
General observation of gels. (**A**) PRP gel. (**B**) 0.25 GO/PRP gel. (**C**) 0.5 GO/PRP gel. (**D**) 1.0 GO/PRP gel. (**E**) PRP gel at 30 s of gelification. (**F**) 1.0 GO/PRP gel at 30 s of gelification

PRP was prepared successfully and it had 1191 ± 35 × 10^9^/l platelets compared to 226 ± 14 × 10^9^/l platelets in the whole blood sample. The pure PRP gel was bright red and the GO/PRP gels were dark red (the higher the GO concentration, the darker the color; Fig. 1A–D). After 30 s of gelification, the PRP gel was not completely formed (Fig. 1E), while all GO/PRP gels had completely formed and were plastic (Fig. 1F).

### Gel characterization

**Figure 2.**
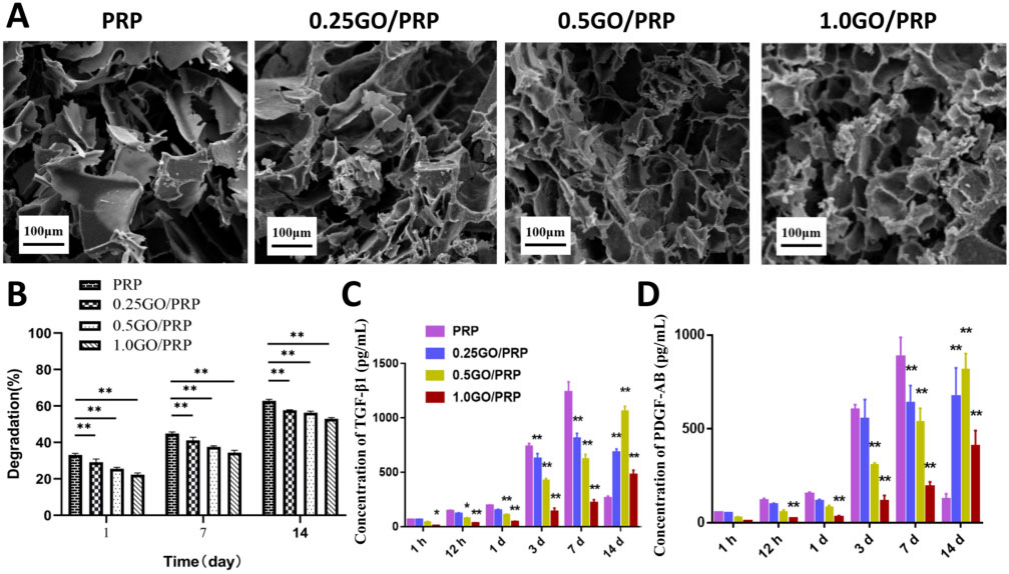
(**A**) SEM images of gels. Scale bar = 100 µm. (**B**) Degradation of gels. (**C**) Release of TGF-β1 at each time point. (**D**) Release of PDGF-AB at each tome point. *P < 0.05 and **P < 0.01 vs PRP

The ultrastructure of each gel was analyzed using SEM. Results showed that all the gels had a porous structure (Fig. 2A). The pore size of the gel with GO was relatively uniform and decreased consistently with the increase in GO concentration (Table 2). The pore size of the PRP gel (107.40 ± 13.91 μm) was significantly higher than that of the 1.0 GO/PRP gel (58.86 ± 9.26 μm; *P* < 0.05). The porosity of the gel also decreased consistently with the increase in GO concentration. The porosity of all gels was > 50%, suggesting an interconnected structure. The compression elastic modulus increased with the increase in GO concentration (Table 2). The elastic modulus of the gels containing GO (0.25 GO/PRP, 2.328 ± 0.054 kPa; 0.5 GO/PRP, 3.255 ± 0.045 kPa; 1.0 GO/PRP, 4.813 ± 0.081 kPa) was significantly higher than that of the PRP gel (0.918 ± 0.030 kPa; *P* < 0.05).

**Table 2.** Characterization of scaffolds

Gel	Pore size (μm)	Porosity (%)	Elastic modulus (kPa)
PRP	107.40 ± 13.91	64.84 ± 0.70	0.918 ± 0.030
0.25GO/PRP	83.69 ± 6.05	63.04 ± 1.00	2.328 ± 0.054
0.5GO/PRP	75.97 ± 5.04	61.08 ± 2.06	3.255 ± 0.045
1.0GO/PRP	58.86 ± 9.26	59.17 ± 2.43	4.813 ± 0.081

During the degradation process, gels were degraded gradually and the degradation accumulation decreased with the increase in GO concentration (Fig. 2B). The sustained release results are shown in Figs 2C and D. During the first 7 days, the PRP gel released most of its TGF-β1 and PDGF-AB, while gels containing GO exerted a controlled release effect on the GFs. The GF release rate decreased with the increase in GO concentration. Over the next 7 days, GFs released from the PRP gel decreased significantly, while the 0.5 GO/PRP and 1.0 GO/PRP gels maintained the release of a large amount of GFs. On the 14th day, the average concentrations of the released TGF-β1 and PDGF-AB from 0.5 GO/PRP gels were 1063.451 ± 44.623 pg/ml and 814.217 ± 86.631 pg/ml, respectively.

### Cell proliferation and viability

**Figure 3.**
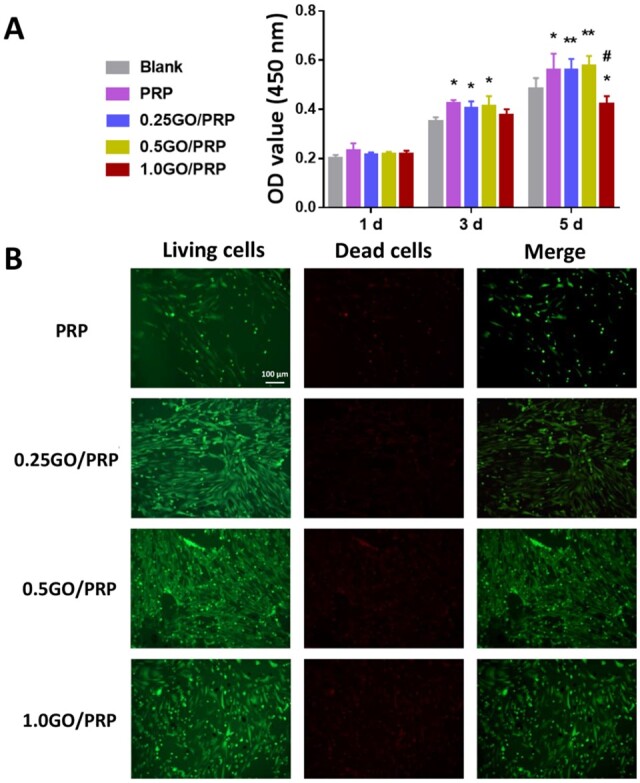
(**A**) Cell proliferation of BMSCs cultured with gels using a CCK-8 kit. (**B**) Images of live–dead staining of BMSCs cultured on gels (green representing living cells and red representing dead cells, scale bar = 100 μm). *P < 0.05 and **P < 0.01 vs blank. ^#^*P* < 0.05 and ^##^*P* < 0.01 vs PRP

After 3 or 5 days of culture, CCK-8 tests were performed. Results showed that there were significantly more cells in the PRP, 0.25 GO/PRP and 0.5GO/PRP groups than in the Blank group (Fig. 3A and S1). The 1.0 GO/PRP group reached the minimum cell number after 5 days of incubation. Live–dead staining was performed to evaluate cell viability (Fig. 3B). After the BMSCs were cultured on each gel for 5 days, all gels supported cell growth. There were more cells on the gels containing GO than on the PRP only gel. However, there were relatively more dead cells on the 1.0 GO/PRP gel compared with the 0.25 GO/PRP or 0.5 GO/PRP gels. These results indicate that the PRP, 0.25 GO/PRP and 0.5 GO/PRP gels had almost no biotoxicity and promoted cell proliferation.

### Cell differentiation

**Figure 4.**
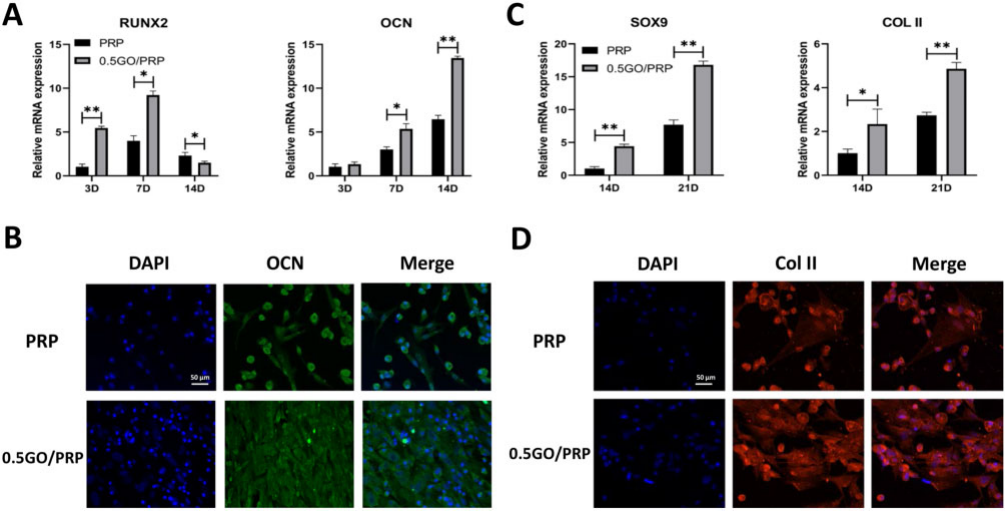
(**A**) The expressions of genes related to osteogenic differentiation analyzed by qRT-PCR. (**B**) Immunofluorescence staining images of OCN expression at 14 d. (**C**) The expressions of genes related to chondrogenic differentiation analyzed by qRT-PCR. (**D**) Immunofluorescence staining images of col II expression at 21 d. Scale bar = 50 μm. *P < 0.05 and **P < 0.01 vs PRP

The above results suggest that the 0.5 GO/PRP gel released GFs continuously, had better mechanical properties than the other gels, maintained cell viability and promoted cell proliferation; therefore, the 0.5 GO/PRP gel was used in follow-up experiments and the PRP gel was used as a control. The osteogenic-related gene expression including *Runx2* and *OCN* was quantified by qRT-PCR at 3, 7 and 14 days of osteogenic induction (Fig. 4A). In the 0.5 GO/PRP group, higher expression of *Runx2* (a marker used for early confirmation of osteogenesis) was found at 3 and 7 days, and higher expression of *OCN* (an indicator of advanced bone maturation) was detected at 14 days. The immunofluorescence staining results showed that the 0.5 GO/PRP group presented more positively stained OCN proteins compared to the PRP group (Fig. 4B). As shown in Figs 4C and D, the 0.5 GO/PRP group also exhibited higher chondrogenic-related gene expression and produced more chondrocyte extracellular matrix (Col II).

### Repairing rotator cuff tears *in vivo*

#### MRI and μCT evaluation

**Figure 5.**
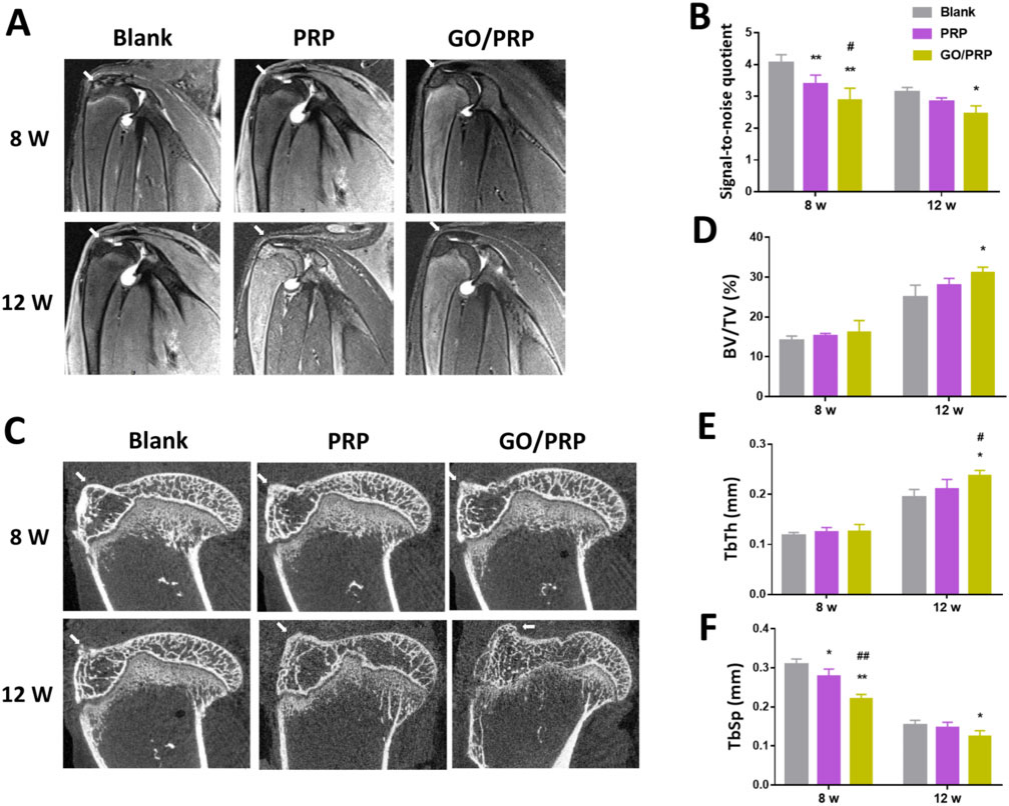
(**A**) MRI images of specimens. (**B**) The average signal-to-noise quotient (SNQ) value. (**C**) μCT results of the specimens. (**D**–**F**) Correlative analysis of new bone formation in the region of interest, (D) bone volume fraction (BV/TV), (E) mean trabecular thickness (TbTh) and (F) mean trabecular spacing (TbSp). The tendon–bone interface is marked by an arrow. *P < 0.05 and **P < 0.01 vs blank. ^#^*P* < 0.05 and ^##^*P* < 0.01 vs PRP

A supraspinatus tendon tear model was used in the current study to assess the gels’ capacity to promote the reconstruction of the TBI area. At 8 and 12 weeks post implantation, MRI scanning was performed (Fig. 5A and S2A). At 8 weeks postoperatively, the supraspinatus tendon at the repair site in the Blank group was partially interrupted and the signal was uneven, while that in the GO/PRP group was continuous and its signal was uniform. At 12 weeks postoperatively, the regenerated tendon in the Blank group was connected to the greater tuberosity; however, it was irregular in shape and there was substantial effusion surrounding the repair area. The signal of the regenerated tendon in the PRP and GO/PRP groups was continuous and uniform without obvious effusion. Signal-to-noise quotient was lowest in the GO/PRP group at both 8 and 12 weeks postoperatively, indicating that the regenerated tendon—including the TBI structure—was more similar to normal tendon tissue (Fig. 5B and Table S1). The μCT scanning and reconstruction results indicate new bone formation at the TBI area was highest in the GO/PRP group at 12 weeks postoperatively: there was obvious bone remodeling, suggesting an increase in bone surface and continuity (Fig. 5C–F and S2B). Furthermore, incomplete cortical bone at the greater tuberosity was observed in Blank group, which had the lowest bone volume fraction and mean TbTh and the highest mean TbSp. At 12 weeks postoperatively, there was no significant difference in the values of BV/TV, TbTh and TbSp between the specimens from normal rabbits and rabbits in the GO/PRP group. These results indicate that GO/PRP promoted bone regeneration at the TBI and enhanced the repair effect on the tendon-bone junction.

#### Histological evaluation

**Figure 6.**
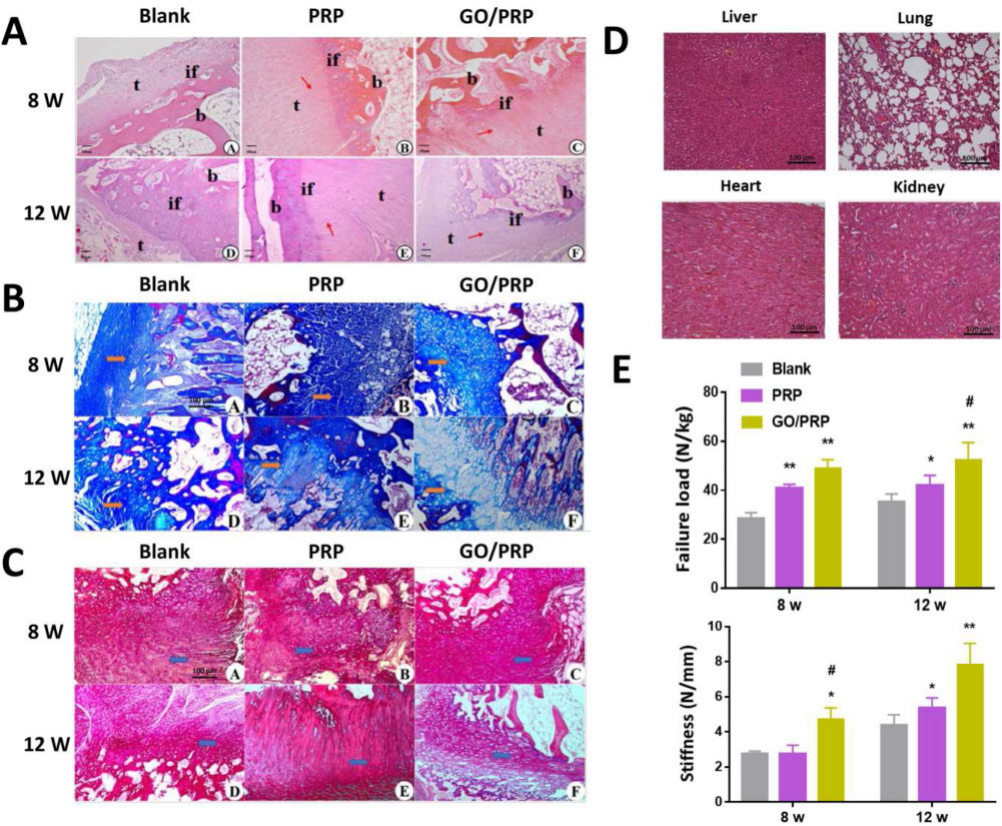
(**A**) Images of H&E staining of specimens. (**B**) Images of Masson’s trichrome staining of specimens. (**C**) Images of picrosirius red staining of specimens. Arrows or if represent tendon–bone interface, t represents tendon, and b represents bone. (**D**) H&E staining images of livers, lungs, hearts and kidneys at 12 weeks post surgery. (**E**) The load to failure and stiffness of specimens. Scale bar = 100 µm. *P < 0.05 and **P < 0.01 vs blank. ^#^*P* < 0.05 and ^##^*P* < 0.01 vs PRP

At 8 and 12 weeks post surgery, specimens in each group were histologically evaluated (Fig. 6A–C). Collagen fiber in the GO/PRP group showed greater continuity and better orientation compared to that in other groups at 8 weeks post implantation. After 12 weeks of surgery, there was obvious fibrocartilage and calcified fibrocartilage with regular distribution in the GO/PRP group, while the newly formed fibrocartilage in the PRP group was irregular and immature, and there were fewer new fibrochondrocytes and disorganized collagen arrangement in the Blank group. Although the regenerated collagen tissue in the PRP group was more organized than that in the Blank group, there were fewer fibrochondrocytes than in the GO/PRP group. The structure of the newly formed TBI tissue in the GO/PRP group was more similar to that of the natural tendon–bone junction. Moreover, no obvious toxic reactions such as blood cell oozing, inflammation or cell necrosis were observed in the results of the H&E staining of internal organs in the GO/PRP group (Fig. 6D). These results indicate that the GO/PRP gel had good biocompatibility and promoted TBI reconstruction and tendon regeneration in the supraspinatus tendon tear model.

#### Biomechanical evaluation

The biomechanical test results at 8 and 12 weeks post surgery were similar, in that the ultimate mean failure load was highest in the GO/PRP group and lowest in the Blank group at both time points (Fig. 6E). The stiffness of the repaired tendon was significantly higher in the GO/PRP group than that in the Blank or PRP groups. Moreover, the failure load and stiffness of the specimens in the GO/PRP group were similar to those from normal rabbits (Table S2). In terms of the failure mode [28], there were three insertional and three midsubstance tears in the Blank group; two insertional and four midsubstance tears in the PRP group; and six midsubstance tears in the GO/PRP group. These results indicate that the repaired tendons in the GO/PRP group had better biomechanical properties and a more stable TBI structure.

## Discussion

The aim of the current study was to confirm that the combination of GO and PRP promoted tendon reconstruction and TBI healing. The 0.5 GO/PRP gel was superior to the other gels in terms of its mechanical properties and good biocompatibility; demonstrated the capacity to promote BMSC proliferation and osteogenic and chondrogenic differentiation; and enhanced the repair effect of the supraspinatus tendon tear in rabbits. These results support our hypotheses and suggest that the 0.5 GO/PRP gel is a promising biomaterial for use in TBI healing and tendon repair.

TBI healing is essential for rotator cuff repair [29]. When the rotator cuff is torn, the surgical operation only reconstructs the integrity of the TBI and cannot restore the TBI structure or biomechanical properties of all involved components, specifically those of the tendon, uncalcified fibrocartilage, calcified fibrocartilage and bone [5, 30]. In addition, the blood supply at the TBI is poor and re-tearing often occurs here [4]. Proper TBI healing is the key to successful tendon repair and is a prerequisite for commencing early rehabilitation exercises. Due to the complex structure of the TBI region, the biomaterials used in tendon repair are required to have a release system for several GFs that promote local cell proliferation and differentiation. PRP, which is rich in a variety of GFs, has aroused considerable interest among researchers [31]. However, there has been some controversy concerning the extent of the effect of PRP on tendon repair, with explanations for the inconsistent results focusing on the different PRP preparation methods and the short half-life of many GFs [14, 32]. Therefore, we incorporated GO, a 2D material widely used in tissue engineering, with the aim of improving the physical and biological properties of PRP and enhancing the repair effect of PRP on TBI healing.

GO has been shown to improve the mechanical properties of gels in terms of meeting the requirements for implantation [33–35]. In this study, the incorporation of GO in a PRP gel improved the organization of the ultrastructure and maintained the interconnected structure, which helped to sustain the exchange of liquids and nutrients and promoted cell growth and differentiation in the local area [36, 37]. The elastic modulus of gels containing GO was also enhanced in line with the increases in GO concentration, which helped to maintain the physical form of the gels and slow down the release of internal GFs. Additionally, GO can be used as a delivery carrier for GFs via physical or chemical bonds [38, 39]. There are a variety of GFs in PRP, and it was hoped that the combination of GO and PRP would create a sustained release system. TGF-β1 and PDGF-AB are the two most abundant factors in PRP [10]. TGF-β1 serves a key function in tissue regeneration, enhancing the formation of collagen, inhibiting macrophage proliferation and promoting stem cell proliferation and osteogenic and chondrogenic differentiation [40–42]. Although there was a burst release in the first day, the gels containing GO sustained the release of TGF-β1 for 14 days, and the controlled release trend in 0.5 GO/PRP and 1.0 GO/PRP did not decrease after 14 days of incubation, suggesting that these gels may continue to release this GF over a longer period. It is thought that excess TGF-β1 may promote the formation of scar tissue; however, this phenomenon was not found in the subsequent *in vivo* experiments. PDGF-AB has been studied only rarely, but its isomer, PDGF-BB, has been shown to promote collagen synthesis and secretion, fibroblast growth and proliferation, and cell chemoattraction and differentiation [8, 11, 43]. The sustained release time of PDGF-AB also exceeded 14 days. Due to the high GO concentration in the 1.0 GO/PRP group, the degradation of the gel was slow and the non-covalent and electrostatic bondings were strong. A large amount of GFs remained in the gel on the 14th day and may be released continuously at high concentrations. The analysis of the GO/PRP gel construction in combination with the results of controlled release results of GFs verified our hypothesis that GO incorporation would improve the physical properties of PRP gels.

As has been demonstrated in numerous studies, GO can promote cell adhesion, proliferation and osteogenic and chondrogenic differentiation [44–46]. The PRP, 0.25 GO/PRP and 0.5 GO/PRP gels significantly promoted BMSC proliferation by 3 and 5 days of culture. The role of GO in promoting cell proliferation was reflected in the differences in cell numbers in the 0.25 GO/PRP and 0.5 GO/PRP groups compared to the PRP group, which were likely due to the larger amount of GFs released from gels with GO compared to those without it. However, when the GO concentration in the gel was increased to 1 mg/ml, a significant decrease in cell number and an increase in dead cells was observed. The biotoxicity of GO in high concentrations has also been shown in previous studies [23, 47]. The inhibition of cell proliferation in the 1.0 GO/PRP group may be caused by the release of a large amount of GO during the degradation of the gel. Therefore, 0.5 GO/PRP gels, with enhanced physical properties and good biocompatibility, were used in the further experiments. Osteogenic differentiation of related cells is essential for TBI healing, and Col II formation caused by chondrogenic differentiation is also the key to TBI reconstruction [5, 48]. After osteogenic or chondrogenic induction, cells cultured on both groups of gels showed osteogenic or chondrogenic differentiation. Although the ratio of stained cells in the immunofluorescence images is similar, there were more OCN formation and Col II deposition in the 0.5 GO/PRP group. Cells cultured on 0.5 GO/PRP gels also showed greater osteogenic- or chondrogenic-related gene expression compared to those cultured on PRP gels. These results indicate that the incorporation of GO indeed promoted BMSC differentiation. Although there is no unified interpretation of the mechanism by which GO promotes cell proliferation and differentiation and thus accelerates tissue repair, many researchers favor the theory that GO continuously improves cell biological behaviors by enriching local nutrients and GFs, while others believe that GO activates certain signaling pathways related to tissue regeneration [49–51]. Further investigation will be required to determine the specific mechanism by which GO works.

Cases of PRP used in clinical treatment or animal surgery for repairing rotator cuff tears have been widely reported [26, 52–54]. The supraspinatus tendon tear model in rabbits was considered to be an appropriate model for simulating human acute rotator cuff tears [28]. The supraspinatus tendon is the most commonly damaged tissue in clinical rotator cuff tears and is mechanically similar in rabbits and humans [55]. Therefore, this animal was chosen in the current study as a model on which to test the repair effect of the GO/PRP gel. In the Blank group, at 12 weeks after surgery, incomplete cortical bone was observed at the greater tuberosity, the structure of the TBI was disorganized and quality of the newly formed tendon was poorest in this group, in terms of its biomechanical properties. In the PRP group, although the newly formed tendon had better biomechanical properties and there was complete tendon–bone connection, the TBI area had a large amount of organized collagen fibers but not enough fibrocartilage. Only the regenerated TBI area in the GO/PRP group had a typical fibrocartilage structure, resulting in a similar morphology and biomechanical properties to those of natural tendons. The MRI results also showed that the newly formed tendon in the GO/PRP group was similar to natural tendon. Additionally, there was no obvious ectopic bone formation in the area surrounding the repair site. The calcification of tendons is one of the main factors that restrict the movement of the shoulder joint after rehabilitation of a rotator cuff tear. As no obvious toxic reactions were observed in the internal organs of the rabbits in the GO/PRP group, the 0.5 GO/PRP gel can be considered a safe and effective treatment of rotator cuff tears.

However, our study has several limitations. First, many clinical rotator cuff tears are chronic cumulative injuries, while the animal model used in this study simulated only acute injuries. Therefore, we recommend that follow-up studies investigate GO/PRP gels in a chronic rotator cuff injury model. Second, the most appropriate GO concentration and the specific mechanism by which GO promotes TBI healing should be further explored. Third, there are multiple GFs in PRP, and only the releases of TGF-β1 and PDGF-AB were quantified in this study. More GFs should be included in future sustained release tests. Fourth, the histological results of newborn tendons were not evaluated quantitatively. In future studies, more abundant histological indexes and quantitative evaluation should be applied. Fifth, there are many types of cells involved in the process of tendon repair, and this study only focused on the effects of gels on the proliferation, osteogenesis and chondrogenesis of BMSCs. Cell behaviors of macrophages, fibroblasts, tenocytes and vascular endothelial cells also need to be further studied. Finally, although the results are encouraging, this research was performed using non-human animal models only. Therefore, preclinical studies with a more meticulous design and larger sample size will be necessary before this treatment can be tested on human participants in a clinical trial.

## Conclusion

In this study, we developed a GO/PRP gel in which GFs are released in a controlled manner. The release of GFs in the 0.5 GO/PRP group could exceed 14 days. The 0.5 GO/PRP gel had a porous and interconnected structure. It was superior to the other gels in terms of its physical properties and biocompatibility. The results of CCK-8, PCR and immunofluorescence staining showed that this gel promoted BMSCs proliferation and osteogenic and chondrogenic differentiation. The results of MRI, μCT, histological evaluation and biomechanical test indicated that the implantation of the 0.5 GO/PRP gel expedited the reconstruction of the torn supraspinatus tendon in a rabbit model by enhancing TBI healing. The newly formed tendon was similar in structure and biomechanical properties to natural tendons. The combination of GO and PRP therefore represents a promising advancement in the treatment of rotator cuff tears.

## Supplementary data

Supplementary data are available at *REGBIO* online.

## Ethics approval and consent to participate

The whole experimental protocol was approved by the Animal Care and Experiment Committee of the Affiliated Traditional Chinese Medicine Hospital of Southwest Medical University (2020680).

## Funding

This work was supported by the Luzhou Municipal People’s Government-Southwest Medical University Science and Technology Cooperation Achievements Transformation Project (2019LZXNYDJ20C01).

*Conflict of interest statement*. None declared.

## Data availability

The raw data required to reproduce these findings are available on reasonable request from the corresponding author (S.F.).

## Supplementary Material

rbab045_Supplementary_Data
