# Correction to: Combination of graphene oxide and platelet-rich plasma improves tendon–bone healing in a rabbit model of supraspinatus tendon reconstruction

**DOI:** 10.1093/rb/rbad034

**Published:** 2023-04-12

This is a correction to: Dingsu Bao, Jiacheng Sun, Min Gong, Jie Shi, Bo Qin, Kai Deng, Gang Liu, Shengqiang Zeng, Zhou Xiang, Shijie Fu, Combination of graphene oxide and platelet-rich plasma improves tendon–bone healing in a rabbit model of supraspinatus tendon reconstruction, *Regenerative Biomaterials*, Volume 8, Issue 6, December 2021, rbab045, https://doi.org/10.1093/rb/rbab045

In the article titled “Combination of graphene oxide and platelet-rich plasma improves tendon-bone healing in a rabbit model of supraspinatus tendon reconstruction”, we notice this following error:

(i) The image in Figure 5A of the PRP group at 8 weeks post surgery was overlaid with the image of the blank group at 12 weeks post surgery when we rearranged the image layout.

The corrected figure is shown herein.

**Figure 5.**
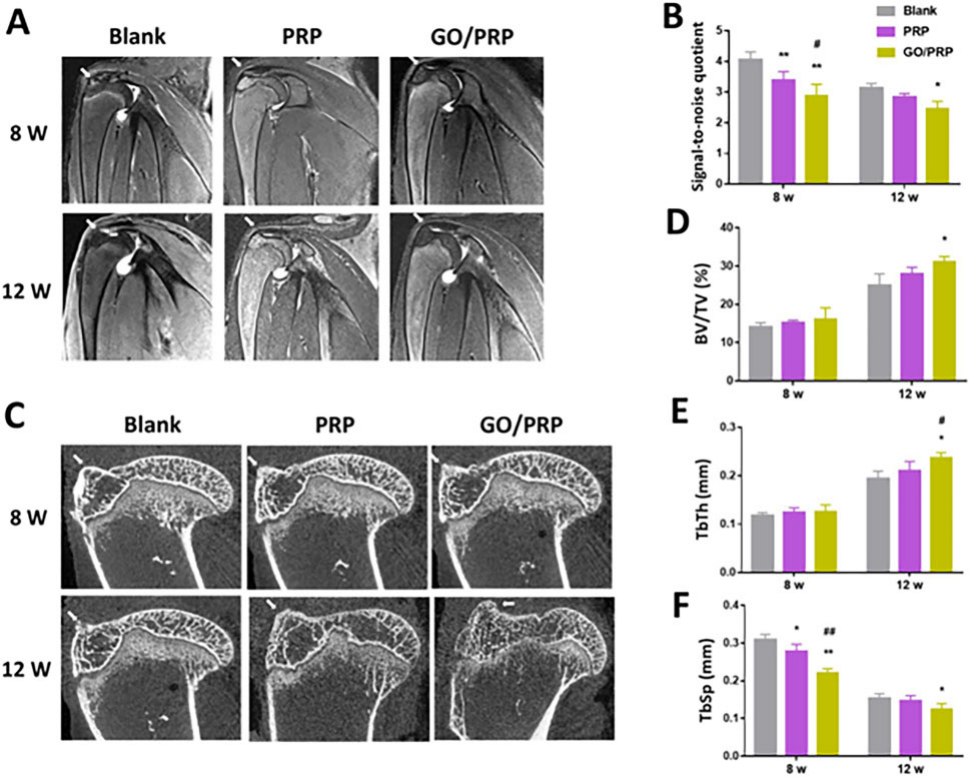
(**A**) MRI images of specimens. (**B**) The average signal-to-noise quotient (SNQ) value. (**C**) μCT results of the specimens. (**D–F**) Correlative analysis of new bone formation in the region of interest, (D) bone volume fraction (BV/TV), (E) mean trabecular thickness (TbTh) and (F) mean trabecular spacing (TbSp). The tendon–bone interface is marked by an arrow. *P < 0.05 and **P < 0.01 vs blank. ^**#**^*P* < 0.05 and ^**##**^*P* < 0.01 vs PRP

