# Supplementary material for: Combination of graphene oxide and platelet-rich plasma improves tendon–bone healing in a rabbit model of supraspinatus tendon reconstruction
Source: Regen Biomater. 2021 Aug 4;8(6):rbab045. doi: 10.1093/rb/rbab045 (PMC8411035; doi:10.1093/rb/rbab045)
Supplement: rbab045_Supplementary_Data [file rbab045_supplementary_data.docx]

**Combination of graphene oxide and platelet-rich plasma improves tendon-bone healing in a rabbit model of supraspinatus tendon reconstruction**

Dingsu Bao^[1, 2, †]^, Jiacheng Sun^[2, †]^, Min Gong^[3]^, Jie Shi^[1]^, Bo Qin^[1]^, Kai Deng ^[1]^, Gang Liu^[1]^, Shengqiang Zeng^[1]^, Zhou Xiang* ^[2]^, Shijie Fu* ^[1]^

^[1]^ *Dr. D. BAO, Dr. J. Shi, Dr. B. Qin, Dr. K. Deng, Dr. G. Liu, Dr. S. Zeng, and Prof. Dr. S. Fu*
Department of Orthopedics, Affiliated Traditional Chinese Medicine Hospital of Southwest Medical University, Luzhou 646000, Sichuan, P. R. China

^[2]^ *Dr. D. BAO, Dr. J. Sun, and Prof. Dr. Z. Xiang*Department of Orthopedics, West China Hospital, Sichuan University, Chengdu 610041, Sichuan, P. R. China

^[3]^ *Dr. M. Gong*Department of Orthopedics, Hospital of Chengdu University of Traditional Chinese Medicine, Chengdu 610075, Sichuan, P. R. China

^[†]^ These two authors contributed equally to this work.

* Corresponding author:

*Prof. Dr. S. Fu,* Department of Orthopedics, Affiliated Traditional Chinese Medicine Hospital of Southwest Medical University, No. 16 Chunhui Road, Luzhou 646000, Sichuan, P. R. China. Email: shijie_fu123@126.com

*Prof. Dr. Z. Xiang,* Department of Orthopedics, West China Hospital, Sichuan University, Guoxue Lane 37, Chengdu 610041, Sichuan, P. R. China. Email: xiangzhou15@hotmail.com


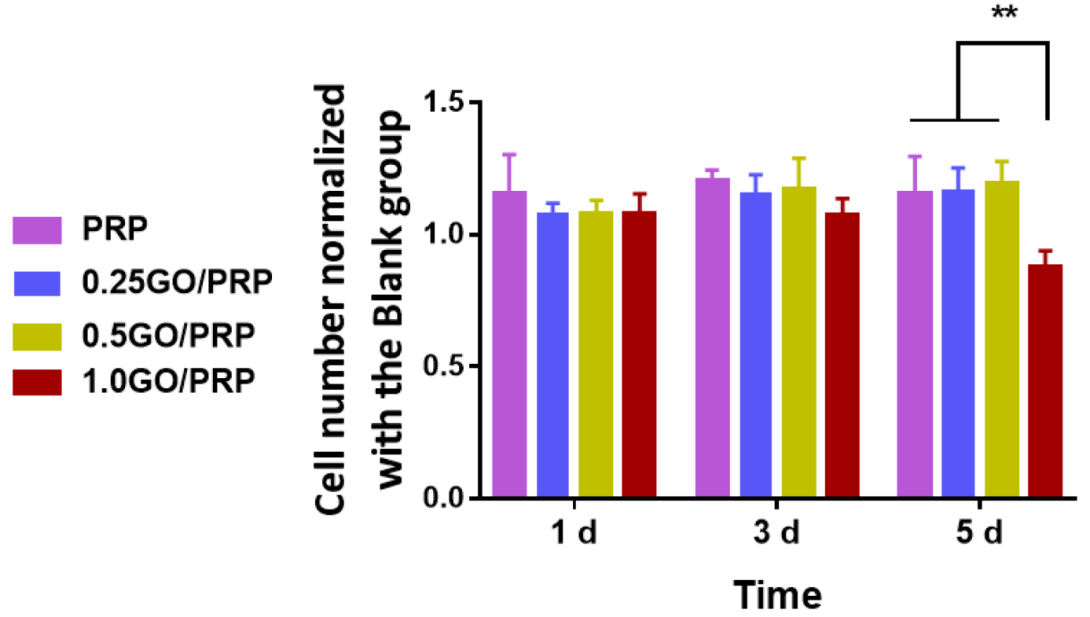


**Figure S1.** Cell proliferation of BMSCs cultured with gels using a CCK-8 kit. Cell numbers were normalized with the Blank group. **p < 0.01 vs 1.0GO/PRP.


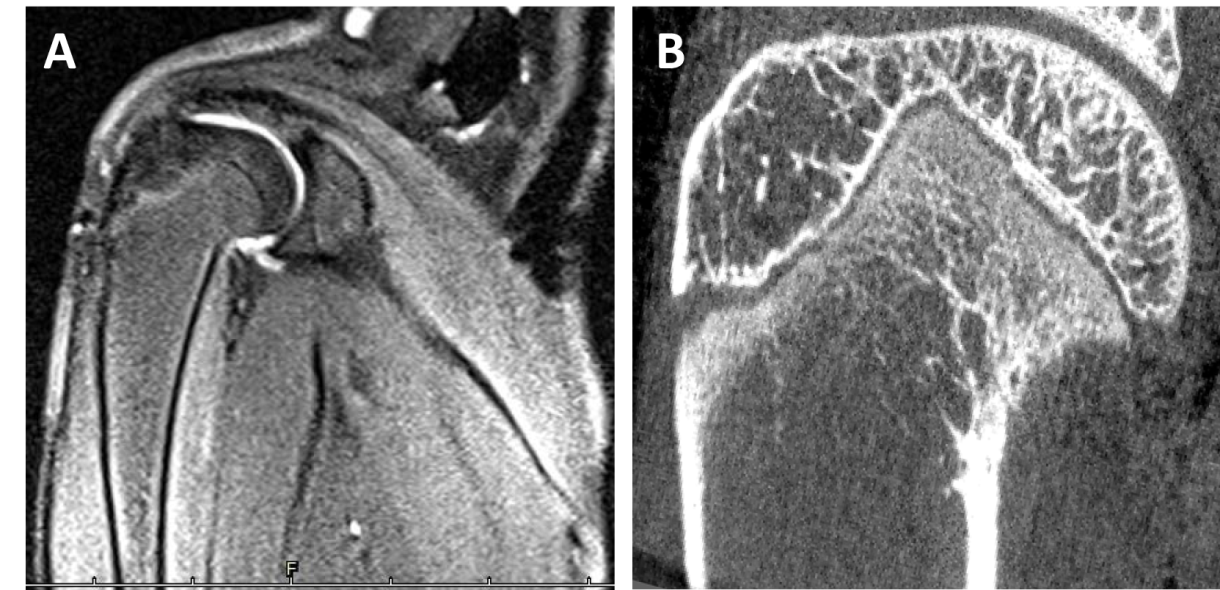


**Figure S2.** (A) MRI image of the normal rabbit. (B) μCT image of the normal rabbit.

**Table S1.** The values of SNQ, BV/TV, TbTh, TbSp, failure load, and stiffness of the specimens from normal rabbits and rabbits in the GO/PRP group (12 w).

|  | Normal | GO/PRP (12 w) |
| --- | --- | --- |
| average signal-to-noise quotient (SNQ) | 2.380 ± 0.100 | 2.460 ± 0.240 |
| bone volume fraction (BV/ TV) (%) | 29.530 ± 0.620 | 31.100 ± 1.400 |
| mean trabecular thickness (TbTh) (mm) | 0.234 ± 0.005 | 0.238 ± 0.010 |
| mean trabecular spacing (TbSp) (mm) | 0.133 ± 0.009 | 0.125 ± 0.014 |
| ultimate mean failure load (N/kg) | 54.127 ± 1.863 | 52.311 ± 7.169 |
| stiffness (N/mm) | 7.964 ± 0.621 | 7.849 ± 1.197 |

There is no significant difference in the values of SNQ, BV/TV, TbTh, TbSp, failure load, and stiffness between the two groups.
